# Efficacy of a dialogic book-sharing intervention in a South African birth cohort: A randomized controlled trial

**DOI:** 10.1016/j.comppsych.2023.152436

**Published:** 2024-01

**Authors:** Sheri-Michelle Koopowitz, Karen Thea Maré, Marilyn Lake, Christopher du Plooy, Nadia Hoffman, Kirsten A. Donald, Susan Malcolm-Smith, Lynne Murray, Heather J. Zar, Peter Cooper, Dan J. Stein

**Affiliations:** a Department of Psychiatry, Neuroscience Institute, Faculty of Health Sciences, University of Cape Town, South Africa; b South African Medical Research Council (SAMRC) Unit on Risk and Resilience in Mental Disorders, Cape Town, South Africa; c Department of Paediatrics & Child Health, Red Cross War Memorial Children's Hospital, and South African Medical Research Council (SAMRC) Unit on Child and Adolescent Health, Cape Town, South Africa; d Department of Psychology, University of Cape Town, South Africa; e School of Psychology and Clinical Language Sciences, University of Reading, Reading, UK

**Keywords:** Parenting, language, Reading, Cognition, Theory of mind

## Abstract

**Objective:**

Evidence shows that dialogic book-sharing improves language development in young children in low-middle income countries (LMICs), particularly receptive and expressive language. It is unclear whether this intervention also boosts development of other neurocognitive and socio-emotional domains in children. Using a randomized controlled trial (RCT) nested in the Drakenstein Child Health Study (DCHS), a book-sharing intervention was implemented in caregivers of 3.5-year-old preschool children living in low-income South African communities.

**Methods:**

122 Caregivers and their children (mean age 3.5 years) were randomly assigned to an intervention group (*n* = 61) or waitlist control group (*n* = 61). A neurocognitive battery determined baseline receptive and expressive language, executive function, theory of mind, and behavior scores.

**Results:**

No differences were observed between intervention and control groups on receptive and expressive language, or any of the neurocognitive or socio-emotional measures from baseline (3.5 years) to 4 months post-intervention administration (4 years).

**Conclusion:**

The benefits noted in prior literature of book-sharing in infants did not appear to be demonstrated at 4 months post-intervention, in children from 3.5 to 4 years of age. This suggests the importance of early intervention and emphasizes the need for further research on adaptation of book-sharing for older participants in a South African context.

**Trial registration**: retrospectively registered on 03/04/2022 **PACTR202204697674974.**

## Highlights

• A book-sharing intervention was administered in a South African birth cohort.
• Neurocognitive and socioemotional variables were measured pre- and post-intervention.
• No differences were found between intervention and control groups on measures.
• Findings highlight the importance of early intervention.

## 1 Introduction

Dialogic book-sharing (DBS) is a parenting reading method used to stimulate reciprocal interactions between young children and their caregivers [1,2]. In randomized controlled trials, the use of book-sharing techniques has been shown to positively contribute to a child's cognitive development, particularly language development [[2], [3], [4]]. Although improvements in different aspects of language have been reported [5], the literature consistently highlights the improvements in both expressive and receptive vocabulary [[6], [7], [8]]. In a meta-analysis of 16 studies, consisting of 626 dyads, relative to controls, the DBS intervention group exhibited significantly greater improvements in expressive vocabulary (*d* = 0.59), as well as in receptive vocabulary (*d* = 0.22) [3]. These vocabulary improvements have been reported, irrespective of the child's age, in samples ranging from ages 14 months to 5 years [[6], [7], [8]]. In addition, book-sharing has been used successfully in low-middle income countries (LMIC) with improvements in expressive and receptive language being observed [7,8]. For example, in a sample of South African infants (between 14 and 16 months at baseline), the intervention group (*n* = 49) exhibited medium increases in lexical production and a large increase of infant comprehension, relative to the control dyads (*n* = 42) [6].

As book-sharing can draw focus to various themes and explore the meaning of events [1,2], initial studies have reported attentional and Theory of Mind (ToM) improvements in DBS intervention groups [6,9]. In a sample of infants, the DBS intervention group (*n* = 49) exhibited an improvement in attention, relative to control dyads (*n* = 42) who did not show a change in attention [6]. Furthermore, in a sample of 4- to 6- year-olds, ToM improvements were evident in children exposed to a greater frequency of parent book-sharing, relative to children with less book-sharing exposure [9]. As Theory of Mind emerges around 4 years of age [10], it is possible that book-sharing at this age would be particularly useful. DBS intervention-linked improvements to potential neurocognitive and socio-emotional gains have been less extensively studied, particularly in a sample of preschool children from impoverished environments where book-sharing is a novel concept.

The aim of the present study was to examine the neurocognitive and socio-emotional effects of a DBS intervention in a sample of LMIC preschool children over a period of six months (3.5–4 years), compared to a waitlist control group receiving care as usual. The impact of dialogic book-sharing on receptive and expressive language, verbal fluency, executive function, attention, and basic theory of mind development of the intervention group was assessed by comparing standardized test scores between the two groups, pre- and post-intervention. We hypothesized that, at post-intervention, the intervention group would perform better than the control group on receptive and expressive language measures, as well as on measures of neurocognitive and socio-emotional development. We are testing whether any improvement in performance among the intervention group between pre- and post-intervention is greater than any improvement in performance seen among the control group.

## 2 Methods

### 2.1 Study design

We conducted a randomized controlled trial (RCT) to evaluate a dialogic book-sharing intervention for parents or caregivers of children aged 3.5 years. This RCT was retrospectively registered with the Pan African Clinical Trials Registry on 03/04/2022 (PACTR202204697674974). This RCT was nested in the Drakenstein Child Health Study (DCHS), an observational cohort study that recruited pregnant women and collected longitudinal data on an array of variables influencing maternal and child health outcomes [[11], [12], [13]]. The DCHS was approved by the Faculty of Health Sciences, Human Research Ethics Committee, University of Cape Town (401/2009) and by the Western Cape Provincial Health Research committee. Specific approval was obtained for the Book-sharing RCT protocol (543/2017) from the Faculty of Health Sciences, Human Research Ethics Committee, University of Cape Town. This study was conducted in accordance with the Declaration of Helsinki.

### 2.2 Study setting

The DCHS recruited participants from two peri-urban relatively stable, low socioeconomic communities (Mbekweni and Paarl East). The former is predominantly an isiXhosa speaking community, and the latter, an Afrikaans speaking community. These communities experience a high prevalence of substance use, exposure to trauma, HIV infection, and poverty [11]. Mothers provided informed consent at enrolment and were re-consented annually. Informed consent was obtained by all dyads in the mother's preferred language: English, Afrikaans, or isiXhosa, before the onset of the study.

### 2.3 Sample selection and size

Pregnant women were recruited from two primary health care clinics for the main DCHS study. Mothers were enrolled at 20 to 28 weeks' gestation while attending routine antenatal care and were prospectively followed. Women were eligible for the study if they were 18 years or older, between 20 and 28 weeks gestation, planned attendance at one of the two recruitment clinics, and intended to remain in the area. During a three-year period (March 2012 to March 2015), 1225 pregnant women were enrolled into the DCHS antenatally; 88 (7.2%) mothers were lost to follow up antenatally, had a miscarriage or a stillbirth. In total, 1137 women gave birth to 1143 live infants (4 twins and 1 triplet).

For the present study, mother-infant dyads recruited by the DCHS were eligible for inclusion if the children were aged 41 to 43 months at the time of baseline assessment. An adult primary caregiver who cohabited with the child was a prerequisite. Twins and/or children with known neurodevelopmental delay or sensory impairment were excluded. A total of 122 eligible caregiver-child dyads were recruited from the relevant age group in the cohort; 61 dyads were randomized into the intervention group and 61 dyads into the waitlist control group, receiving care as usual.

### 2.4 Measures

All measures were administered in the child's home language as all measures were translated into Afrikaans and isiXhosa.

#### 2.4.1 Language

Receptive and expressive language was measured using the Peabody Picture Vocabulary Test, fourth edition (PPVT-4) [14] and KABC-II Expressive Vocabulary [15] subtests respectively. The PPVT-4 asks the child to choose the corresponding picture based on a verbal prompt. The PPVT-4 has been translated into both Afrikaans and isiXhosa [16]. This measure has been used in book-sharing RCTs in low-income South African communities [17]. The KABC-II Expressive Vocabulary task measures the child's ability to correctly name a set of pictures. This measure has been used in children from low-income South African communities [18].

#### 2.4.2 Selective attention

The Balloon Hunt Task is a paper-and-pencil task from TEA-Ch2 J (5–7 years) designed to evaluate visual inattention [19], and has been used successfully in randomized controlled trials [20]. The test consists of two subtests: subtest A requires the child to find 20 circles with an attached vertical line (‘balloons’) among a much larger number of circles that act as distractors. Each trial lasts 10 s. Subtest B requires serial search for 20 circles among many balloons and has no time limit.

#### 2.4.3 Executive function

Working memory was assessed using the Picture Memory task from the Wechsler Preschool and Primary Scale of Intelligence, fourth edition (WPPSI-IV) [21]. The Picture Memory subtask uses the familiarize-recognize paradigm, in which the child has a few seconds to memorize a picture (i.e. a book, gift, or clover leaf) that then has to be recognized from a set of one or more dissimilar distractors [21]. The Picture Memory task from the WPPSI-IV battery has been successfully used in South African preschool children, as well as book-sharing interventions [7,22].

The Dimensional Change Card Sort (DCCS) [23,24], which resembles the Wisconsin Card Sorting Test (WCST), measured cognitive flexibility. Participants are required to sort cards with two salient features/dimensions (e.g., shape or color), first according to one dimension and then according to the other. This measure has been successfully used in a South African, low-income sample of 3 to 5 year old children [25].

The adapted Stroop-like day-night task assesses the child's ability to inhibit an automatic response. In the control trials, the child is required to say “dog” when shown the dog card, and “banana” when shown the banana card. In the conflict condition, the child was required to say the opposite of what is shown on a set of cards (children were instructed to say “dog” when presented with the banana card, and vice versa). There were 2 practice trials and 10 test trials for each condition. The cards were presented in the same order across the sample [26,27]. This Stroop task has been used in samples as young as 4 years of age [26].

#### 2.4.4 Social cognition

Theory of Mind was examined using Diverse Desires and Diverse Beliefs tasks from the early and basic modules of the UCT Theory of Mind battery [[28], [29], [30]]. The Diverse Desires task assesses the ability to understand that a character can have desires different from one's own, and that these desires will influence the choices others make. Similarly, the Diverse Beliefs task assesses the ability to understand that a character can have beliefs different from one's own, and that these beliefs will influence a character's actions. The Diverse Desires task was administered both at baseline and post-intervention, however the Diverse Beliefs task was administered only at 4 years since this is the youngest age children can reasonably be expected to attempt this test [30]. These tasks have been successfully used in South African child research settings [28,29].

#### 2.4.5 Internalizing and externalizing behavior

The Child Behavior Checklist (CBCL for children aged 1.5–5 years) [31] is a parent report questionnaire that measures the presence of internalizing and externalizing behaviors in the last 6 months. Ninety-nine items ask about these behaviors using a 3-point Likert scale. We used the scores for Internalizing and Externalizing behavior subscales. This measure has been used in low-income South African community research [18].

### 2.5 Procedures

#### 2.5.1 Study procedures

Consecutive children attending the standard 3.5-year psychosocial assessment of the DCHS were recruited by DCHS fieldworkers for the book-sharing intervention. After enrolment, block randomization to an intervention or control group which was completed off-site. Post-randomization confirmation was obtained that the gender of the children was evenly distributed among both groups. Outcome measures were collected at the 3.5-year psychosocial DCHS visit (baseline for the present study), and post-intervention 6 months later (4 years). Following the 3.5-year visit, the intervention group underwent the book-sharing intervention. The DCHS design allows only a few visits per year to reduce participant burden, the post-intervention assessments were scheduled for the 4-year psychosocial visit. During the pre- and post-intervention assessments, where needed, isiXhosa translators were used during assessments. Numerous breaks were given between tasks to ensure that the child did not get too tired, as assessments took approximately one hour to complete. Participants were reimbursed for travel expenses.

#### 2.5.2 Randomization and blinding

This parallel intervention used permuted block randomization. Group allocation was determined by the holder of the sequence who was situated off-site. Participants were allocated to the intervention group (*n* = 61) or to the control group (*n* = 61). Outcome assessors were blinded to group status, whereas participants and fieldworkers were not blinded to group status.

#### 2.5.3 Intervention program

The intervention commenced in April 2018 and ran to completion in December 2019. The book-sharing program is a group-based parenting intervention based on previous programs implemented and investigated in similar settings in South Africa [17,32]. The intervention consisted of 90-min weekly sessions for eight consecutive weeks. The book-sharing intervention was conducted in the participants' first language, where two trained facilitators, one isiXhosa speaking and one Afrikaans speaking, led the groups. The program was delivered to groups of four to six parent/caregivers in local libraries. Each session consisted of a PowerPoint presentation with information on techniques and demonstration videos. The skills build on each other with progressively more complex concepts explored (see Table 1). Caregivers were given the opportunity to practice book-sharing with their child with one-on-one supervision provided. The sessions were designed to be interactive and positive so as to model the desired interaction between caregiver and child when book-sharing. For the first seven sessions there was a ‘book of the week’ (see Table 1) where the families were given books to keep and encouraged to practice book-sharing at home for 10–15 min per day. Participants were given laminated memory cards with the week's lessons and examples to collect in a provided file. In the final session, all the key principles were reviewed, and caregivers registered for library cards. Participants were encouraged to continue practicing their skills for 10–15 min per day with new books obtained from the library, second-hand bookshops or borrowed from each other. The control group received services as usual (attending the regular DCHS visits and interacting with their children as usual) and were added to a waiting list to receive the intervention should the results prove favorable.

**Table 1:** Weekly themes of intervention sessions with accompanying books.

Week	Theme/Skill	Book of the week
1	Pointing and Naming – language enrichment; building vocabulary	Handa's Surprise⁎⁎
2	Making Links – to the child's everyday experiences and values	Little Helpers⁎
3	Numbers and Comparisons – counting and noticing in-group differences	Handa's Hen⁎⁎⁎
4	Emotions – identifying and mimicking facial expressions, body language	Hug⁎
5	Intentions – people have specific aims with their behaviors that may differ from yours	The Herd Boy⁎⁎
6	Perceptions – you may have different ideas/opinions depending on what you observe/experience	Yawning is catching⁎⁎
7	Relationships – conflict arises in healthy relationships and can be resolved	All's well that ends well⁎
8	Revision of all key concepts and skills	Library Card application

⁎ Denotes books with pictures only.

⁎⁎ Books are available in isiXhosa and Afrikaans and were provided in these languages.

⁎⁎⁎ Book only available in English.

The interventionists, who were first language speakers of the languages the intervention was administered in, were trained in book-sharing methods by The Mikhulu Trust (https://www.mikhulutrust.org). Training consisted of 5 sessions, lasting approximately 2 h each, during which the principles of the intervention were taught and practiced. The interventionists had weekly supervision meetings following the group sessions to discuss any difficulties delivering the intervention.

Intervention adherence was determined using participant feedback. The sessions were conducted on the same day, time, and place every week. The interventionists made telephonic contact with participants once a week to remind them of the next session. At the beginning of each session, participants were asked to provide feedback regarding their home book-sharing experiences. 69% of the intervention participants attended at least 6 out of 8 intervention sessions.

### 2.6 Data analysis

Intervention and control groups were first compared on social, maternal and child factors at baseline to assess efficacy of the randomization method in producing equivalent groups, using means and standard deviations for continuous variables and frequencies with corresponding percentages for categorical variables. Chi-squared tests (and Fisher tests for infrequent cell counts) were used to compare groups on categorical variables, whilst *t*-tests (and Mann-Whitney-Wilcoxon tests where assumption/s were violated) were utilized for continuous variables. Baseline performance of intervention and control groups was compared for all major neurocognitive, in order to confirm to what extent groups demonstrated similar functioning at baseline.

We ran intention-to-treat (ITT) analyses and included all participants who were enrolled in the RCT, even if they dropped out of the program. A series of complete-case linear mixed-effect models (LMM) using restricted maximum likelihood estimation were conducted in *R* using the *lme4* package [33], each with an added interaction term to investigate whether intervention and control groups differed in performance on various child neurocognitive and socio-emotional measures across pre- and post-intervention sessions. Each of the mixed-effect models incorporated a random intercept, which accounted for within-individual dependency in performance over time, and thus allowed for the estimation of any fixed effect that may emerge from overall performance differences between intervention and control groups. In the case of the Diverse Desires task, which is represented as a dichotomous variable, a logistic mixed-effects model was conducted. For each of the specified models where there was a significant interaction effect, defined by a *p* < 0.05, intervention and control groups were considered to differ in performance between pre- and post- intervention sessions, and effects were investigated further to better determine the nature and direction of the effect. Standardized coefficients were used as a measure of effect size for continuous outcomes, while odd ratios were used as a measure of effect size when outcomes were binary.

For measures that were only conducted at the post-intervention session, including the Balloons Task, a simple linear regression was run to compare performance between intervention and control groups. Conversely, for the DCCS and Diverse Beliefs tasks, given the dichotomous nature of scoring, logistic regression models were run.

## 3 Results

### 3.1 Intervention and control group baseline comparison

The descriptive statistics indicate that the intervention (*n* = 61) and the control group (n = 61) were equivalent across all social, maternal and child factors (see Table 2). When comparing the intervention group with those who were not enrolled in the intervention (*n* = 1027), a higher proportion of mothers with depression were recruited for the intervention.

**Table 2:** Demographic variables (social, maternal and child factors).

	Intervention	Control	*p* value	Remaining birth cohort	*p* value^+^
	(N = 61)	(*N* = 55)		(N = 1027)	
**Child variables**					
Site			0.50		0.50
Mbekweni: TC Newman	31:30 (50.8%:49.2%)	31:24 (56.4%:43.6%)		572:455 (55.7%:44.3%)	
Child Gender			0.50		0.70
Female:Male	31:30 (50.8%:49.2%)	31:24 (56.4%:43.6%)		495:532 (48.2%:51.8%)	
Child Age			0.50		0.075
Mean (SD)	3.50 (0.05)	3.52 (0.04)		3.49 (0.05)	
Child exposure to community violence (CECV)^2^			0.07		0.80
Mean (SD)	3.00 (3.87)	1.60 (2.59)		3.46 (3.92)	
General cognition (KABC NVI)			0.40		
Mean (SD)	76.6 (14.8)	78.6 (15.3)		76.8 (14.7)	0.90
**Maternal variables**					
Maternal education			0.20		0.30
Lower than secondary education	34 (55.7%)	26 (47.3%)		635 (61.8%)	
At least secondary education	27 (44.3%)	29 (52.7%)		392 (38.2%)	
Socioeconomic status			0.06		0.90
lowest SES	16 (26.2%)	7 (12.7%)		285 (27.8%)	
low-mod SES	14 (23.0%)	13 (23.6%)		237 (23.1%)	
mod-high SES	15 (24.6%)	15 (27.3%)		261 (25.5%)	
high SES	16 (26.2%)	20 (36.4%)		241 (23.5%)	
Depression (BDI)			0.30		
Probable moderate/severe clinical	8 (13.1%)	4 (7.3%)		40 (5.8%)	0.044*
Probable sub-threshold	50 (82.0%)	46 (83.6%)		645 (94.2%)	
Lifetime emotional abuse (IPV)^1^			0.06		0.60
No IPV	35 (57.4%)	31 (56.4%)		409 (61.2%)	
Isolated incident	5 (8.2%)	1 (1.8%)		50 (7.5%)	
Low frequency	5 (8.2%)	2 (3.6%)		28 (4.2%)	
Mild frequency	8 (13.1%)	12 (21.8%)		117 (17.5%)	
High frequency	5 (8.2%)	4 (7.3%)		64 (9.6%)	
Lifetime physical abuse (IPV)^1^			0.07		0.061
No IPV	30 (49.2%)	31 (56.4%)		417 (62.4%)	
Isolated incident	7 (11.5%)	7 (12.7%)		66 (9.9%)	
Low frequency	11 (18.0%)	3 (5.5%)		53 (7.9%)	
Mild frequency	8 (13.1%)	7 (12.7%)		82 (12.3%)	
High frequency	2 (3.3%)	2 (3.6%)		50 (7.5%)	
Lifetime Sexual abuse (IPV)^1^			0.30		0.12
No IPV	49 (80.3%)	44 (80.0%)		597 (89.4%)	
Isolated incident	4 (6.6%)	1 (1.8%)		20 (3%)	
Low frequency	2 (3.3%)	2 (3.6%)		5 (0.7%)	
Mild frequency	2 (3.3%)	3 (5.5%)		29 (4.3%)	
High frequency	1 (1.6%)	0 (0%)		17 (2.5%)	
Risk of alcohol use disorder (ASSIST)^1^			0.40		0.11
Lower risk	45 (73.8%)	42 (76.4%)		551 (85.2%)	
Moderate risk	12 (19.7%)	7 (12.7%)		74 (11.4%)	
High risk	1 (1.6%)	1 (1.8%)		22 (3.4%)	
Risk of tobacco use disorder (ASSIST)^2^			0.40		0.50
Lower risk	40 (65.6%)	38 (69.1%)		472 (72.8%)	
Moderate risk	17 (27.9%)	11 (20.0%)		168 (25.9%)	
High risk	1 (1.6%)	1 (1.8%)		8 (1.2%)	
Household perceived food insecurity^1^			0.10		0.50
Perceived food insecure	7 (11.5%)	3 (5.5%)		66 (9.6%)	
Perceived food secure	48 (78.7%)	48 (87.3%)		620 (90.4%)	

Note: * p < 0.05; ^1^Fisher-test conducted; ^2^Mann-WhitneyWilcoxon test conducted; ^+^Comparison of intervention group relative to the remaining birth cohort who were not enrolled.

### 3.2 Intervention results

Table 3 highlights the outcome measures for pre- and post-intervention. At post- from pre-intervention, both the intervention and control groups demonstrated significant improvements in expressive vocabulary (*t* = 2.57, *df* = 98.27, *p* = 0.01, β = 0.37), working memory (*t* = 2.57, *df* = 97.03, *p* = 0.01, β = 0.41), inhibitory control (*t* = 3.04, *df* = 85.75, *p* = 0.002, β = 0.63), and socio-emotional functioning (*OR* = 3.20, *df* = 189, *p* = 0.016). In addition, both groups demonstrated greater internalizing (*t* = 6.70, *df* = 113.25, *p* < 0.001, β = 1.01) and greater externalizing (*t* = 4.48, *df* = 112.17, *p* < 0.001, β = 0.71) behaviors at the post-intervention assessment (see Table 4).

**Table 3:** Outcome measures for intervention and control groups at pre- and post-intervention.

	Pre-Intervention		Post-intervention	
Variables	Intervention group	Control	Intervention group	Control
Expressive vocabulary (KABC)	10.3 (2.1)	10.9 (2.4)	11.4 (2.2)	11.6 (2.7)
Picture memory (PPVT-4)	25.2 (8.4)	26.3 (10.3)	28.2 (9.1)	29.6 (10.7)
Picture vocabulary (WPPSI)	4.2 (2.6)	4.9 (3.6)	5.9 (3.4)	5.6 (3.9)
Inhibitory control (Day/night Stroop)	3.0 (3.8)	3.6 (4.2)	6.2 (3.8)	6.2 (3.9)
Diverse desires [Fail, Success]	[17, 33]	[20, 21]	[21, 33]	[13,35]
Internalizing (CBCL)	3.7 (4.9)	4.5 (4.8)	13.1 (7.5)	12.2 (7.5)
Externalizing (CBCL)	6.3 (6.4)	7.8 (8.3)	13.5 (6.5)	13.6 (8.8)
Diverse beliefs [Fail, Success]	–	–	1.2 (0.9)	1.2 (0.8)
Visual attention (Balloons)	–	–	22.0 (6.7)	22.5 (7.3)
Cognitive flexibility (DCCS) [Fail, Success]	–	–	[44,7]	[44, 5]

**Table 4:** Treatment effect estimates across 10 outcomes.

			Main effect 1			Main effect 2		Interaction effect		
Outcome model		Intervention group			Post-intervention					
	Intercept (B)	β [95% CI]	B [95% CI]	*p* value	β [95% CI]	B [95% CI]	*p* value	β [95% CI]	B [95% CI]	*p* value
M1: Expressive vocabulary (KABC)	10.70 [10.02:11.38]	−0.21 [−0.60:0.17]	−0.51 [−1.43:0.42]	0.279	0.37 [0.09:0.65]	0.88 [0.21:1.56]	0.011*	0.11 [−0.28:0.50]	0.26 [−0.66:1.18]	0.578
M2: Picture memory (PPVT-4)	25.93 [23.14:28.73]	−0.13 [−0.53:0.26]	−1.29 [−5.11:2.53]	0.506	0.39 [0.09:0.69]	3.80 [0.88:6.72]	0.011*	−0.05 [−0.46:0.37]	−0.48 [−4.50:3.55]	0.816
M3: Picture vocabulary (WPPSI)	4.74 [3.81:5.67]	−0.19 [−0.58:0.20]	−0.60 [−1.87:0.66]	0.346	0.41 [0.09:0.73]	1.32 [0.28:2.36]	0.013*	0.22 [−0.22:0.66]	0.70 [−0.73:2.13]	0.334
M4: Inhibitory control (Day/night Stroop)	3.57 [2.06:5.09]	−0.18 [−0.68:0.31]	−0.76 [−2.77:1.26]	0.461	0.63 [0.22:1.05]	2.60 [0.91:4.29]	0.003*	0.19 [−0.36:0.75]	0.79 [−1.50:3.07]	0.497
M5: Diverse desires^2^	0.95 [0.49:1.82]	−0.14 [−0.28:0.00]	0.53 [0.21:1.30]	0.162	0.26 [0.12:0.40]	3.04 [1.18:7.85]	0.021*	−0.12 [−0.30:0.06]	1.07 [0.31:3.67]	0.913
M6: Internalizing (CBCL)	4.52 [2.84:6.20]	−0.11 [−0.41:0.19]	−0.83 [−3.14:1.47]	0.477	1.01 [0.71:1.31]	7.66 [5.41:9.91]	<0.001*	0.23 [−0.17:0.64]	1.76 [−1.30:4.82]	0.258
M7: Externalizing (CBCL)	7.86 [5.85:9.87]	−0.19 [−0.53:0.15]	−1.56 [−4.32:1.19]	0.264	0.71 [0.40:1.02]	5.79 [3.24:8.34]	<0.001*	0.17 [−0.26:0.59]	1.37 [−2.10:4.83]	0.438
M8: Diverse beliefs^1,2^	0.66 [0.36:1.16]	0.10 [−0.09:0.30]	1.53 [0.70:3.38]	0.292	-	–	–		–	–
M9: Visual attention (Balloons)	22.46 [20.50:24.40]	−0.07 [−0.46:0.32]	−0.48 [−3.21:2.25]	0.731		–	–		–	–
M10: Cognitive flexibility (DCCS)^1,2^	0.11 [0.04:0.26]	0.03 [−0.10:0.15]	1.31 [0.39:4.71]	0.664	-	–	–		–	–

*Note: β* = Standardized coefficient (Effect size when outcome is continuous). B = Unstandardized coefficient. ^1^ Conducted at post-intervention only. ^2^ Risk differences (Effect size when outcome is dichotomous) and odds ratios presented respectively.

The intervention group demonstrated equivalent performance on receptive and expressive language, as well as equivalent performance on other neurocognitive and socio-emotional, relative to the waitlist control group.

## 4 Discussion

Book-sharing was no more effective than normal development in the waiting list control on receptive and expressive language, as well as neurocognitive and socio-emotional measures, at post-intervention. Both groups improved, as expected, with age when comparing test scores 6 months later.

Contrary to individual studies reporting improvements across age ranges [[6], [7], [8]], we were unable to detect significant group differences between the intervention group and controls on receptive and expressive language, cognitive, and socio-emotional measures in this age group. There are some potential explanations. First, some evidence suggests that book-sharing does not improve language past a certain age; a meta-analysis reported that in the 4- to 5-year old group, the intervention group did not exhibit language improvements following a dialogic book-sharing intervention [3]. There may be a window of time for maximum impact for there to be observable language, cognitive, and socio-emotional improvements. As most dialogic book-sharing studies with positive findings have been conducted on younger samples (14 months to 36 months), it is possible that our sample (3.5–4 years at the time of the intervention) may be just outside of the cut off for neurocognitive and socio-emotional effects to be detected. Second, a meta-analysis highlighted that smaller effects sizes have been reported in children who were at risk of poor language and/or cognitive development [3]. It is therefore relevant that the studied communities experience a high prevalence of substance use, exposure to trauma, HIV infection, and poverty [11]. Furthermore, it may be that in these particular communities, where more serious matters (such as food security and violence) take precedence, book-sharing was a low priority for participants. This is supported by a meta-analysis that highlighted that participants exposed to high levels of adversity are less likely to use book-sharing methods, relative to more advantaged participants [4]. Third, the follow up assessment was scheduled for the next DCHS visit, 4 months post-intervention. It is possible that evaluation immediately after the trial may have showed some effect. Nevertheless, participants were encouraged to continue their book-sharing efforts once the intervention concluded. Notably, there are few studies that measure the effects of a book-sharing intervention months after the conclusion of the intervention [34].

A number of limitations to our study deserve emphasis. First, the sample size was relatively small. However, our sample size is similar to those of previously published DBS studies. Future studies should include larger sample sizes to accurately examine the effects of a book-sharing intervention on receptive and expressive language in older preschool children. Second, at times, assessments were conducted with the use of interpreters; ideally the assessor should be fluent in the child's first language. Third, this book-sharing intervention was relatively short to expect noticeable non-language neurocognitive and socio-emotional variables. An intervention longer in duration may be beneficial in samples where reading and book-sharing are a novel concept as it may help with acceptance and uptake of the intervention homework. Finally, as noted above, the post-intervention assessment was conducted 4 months after the conclusion of the intervention. The null findings may be indicative of failed maintenance efforts rather than the intervention itself.

## 5 Conclusions

In children aged 3.5 years in a LMIC country setting, dialogic book-sharing did not improve language proficiency (receptive and expressive language), or neurocognitive and socio-emotional gains at 4 months post-intervention. Possible reasons for these findings include that there may be a critical period in which to implement book-sharing interventions to produce noticeable neurocognitive and socio-emotional differences. The findings suggest the importance of early intervention and emphasizes the need for further research on adaptation of book-sharing for older participants in a South African context.

## Trial registration

This trial was retrospectively registered on the Pan African Clinical Trial Registry on 03/04/2022; PACTR202204697674974.

## Ethics approval and consent to participate

Specific approval was obtained for the Book-sharing RCT protocol (543/2017) from the Faculty of Health Sciences, Human Research Ethics Committee, University of Cape Town. Mothers provided informed consent at enrolment and yearly after that. Informed consent was obtained for all participants in their preferred language: English, Afrikaans, or isiXhosa, before the onset of the study. The DCHS was approved by the Faculty of Health Sciences, Human Research Ethics Committee, University of Cape Town (401/2009) and by the Western Cape Provincial Health Research committee. This study was conducted in accordance with the Declaration of Helsinki.

## Competing interests

The intervention was conceptualized by PC and DJS, with guidance from LM and HJZ. All other authors declare no conflicts of interest.

## Declaration of interest

DJS and HJZ are funded by the South African Medical Research Council (SAMRC).

## Funding

The RCT was conducted with no additional funding beyond what the DCHS already received. Support for the DCHS was provided by the 10.13039/100000865Bill and Melinda Gates Foundation (Grant OPP1017641), the National Institute of Mental Health (Grant 1R21MH098662–01), the 10.13039/501100001321National Research Foundation and the 10.13039/501100001322South African Medical Research Council.

## Data Availability

The Drakenstein Child Health Study is committed to the principle of data sharing. De-identified data will be made available to requesting researchers as appropriate. Requests for collaborations to undertake data analysis are welcome. More information can be found on our website [http://www.paediatrics.uct.ac.za/scah/dclhs].
